# Nimodipine Protects Vascular and Cognitive Function in an Animal Model of Cerebral Small Vessel Disease

**DOI:** 10.1161/STROKEAHA.124.047154

**Published:** 2024-06-11

**Authors:** Zhiyuan Yang, Frédéric Lange, Yiqing Xia, Casey Chertavian, Katerina Cabolis, Marija Sajic, David J. Werring, Ilias Tachtsidis, Kenneth J. Smith

**Affiliations:** 1 Department of Neuroinflammation, UCL Queen Square Institute of Neurology (Z.Y., Y.X., C.C., K.C., M.S., K.J.S.), University College London, United Kingdom.; 2 Department of Medical Physics and Biomedical Engineering (F.L., I.T.), University College London, United Kingdom.; 3 Stroke Research Centre, Department of Brain Repair and Rehabilitation, UCL Queen Square Institute of Neurology (D.J.W.), University College London, United Kingdom.

**Keywords:** calcium channel blockers, cerebrovascular circulation, neurovascular coupling, open field test, rats, inbred SHR, spectroscopy, near-infrared

## Abstract

**BACKGROUND:**

Cerebral small vessel disease is a common cause of vascular cognitive impairment and dementia. There is an urgent need for preventative treatments for vascular cognitive impairment and dementia, and reducing vascular dysfunction may provide a therapeutic route. Here, we investigate whether the chronic administration of nimodipine, a central nervous system-selective dihydropyridine calcium channel blocking agent, protects vascular, metabolic, and cognitive function in an animal model of cerebral small vessel disease, the spontaneously hypertensive stroke-prone rat.

**METHODS:**

Male spontaneously hypertensive stroke-prone rats were randomly allocated to receive either a placebo (n=24) or nimodipine (n=24) diet between 3 and 6 months of age. Animals were examined daily for any neurological deficits, and vascular function was assessed in terms of neurovascular and neurometabolic coupling at 3 and 6 months of age, and cerebrovascular reactivity at 6 months of age. Cognitive function was evaluated using the novel object recognition test at 6 months of age.

**RESULTS:**

Six untreated control animals were terminated prematurely due to strokes, including one due to seizure, but no treated animals experienced strokes and so had a higher survival (*P*=0.0088). Vascular function was significantly impaired with disease progression, but nimodipine treatment partially preserved neurovascular coupling and neurometabolic coupling, indicated by larger (*P*<0.001) and more prompt responses (*P*<0.01), and less habituation upon repeated stimulation (*P*<0.01). Also, animals treated with nimodipine showed greater cerebrovascular reactivity, indicated by larger dilation of arterioles (*P*=0.015) and an increase in blood flow velocity (*P*=0.001). This protection of vascular and metabolic function achieved by nimodipine treatment was associated with better cognitive function (*P*<0.001) in the treated animals.

**CONCLUSIONS:**

Chronic treatment with nimodipine protects from strokes, and vascular and cognitive deficits in spontaneously hypertensive stroke-prone rat. Nimodipine may provide an effective preventive treatment for stroke and cognitive decline in cerebral small vessel disease.

Cerebral small vessel disease (cSVD) describes disorders that affect the arterioles, capillaries, and venules of the brain,^1^ which account for about 1 in 3 ischemic strokes, 80% of strokes due to intracerebral hemorrhage,^2^ and contribute to up to 45% of all cases of dementia.^3^ The mechanisms by which vascular pathology develops into vascular cognitive impairment and dementia remain uncertain, impeding the development of effective treatments. Indeed, current management of cSVD, which focuses on controlling risk factors (such as hypertension), shows inconsistent outcomes in cognitive protection and limited efficacy.^4,5^ Early treatment is likely to be most beneficial, raising the need for biomarkers predictive of later cognitive decline.

The vascular dysfunction associated with cSVD has emerged as a promising biomarker of forthcoming cognitive decline. Apart from cerebral hypoperfusion,^6^ there are important deficits in dynamic vascular function, namely neurovascular coupling (NVC)^7^ and cerebrovascular reactivity (CVR),^8^ mainly due to increased arterial stiffness and endothelial dysfunction.^9^ In fact, it is the deficit in dynamic function of the vasculature, rather than in cerebral perfusion at resting state, which best predicts clinical outcomes,^10^ including poor cognitive performance.^11^ Vascular dysfunction impairs cognition, probably through mechanisms including hypoxic neuronal damage,^12^ amyloid deposition (as in the 2-hit hypothesis of Alzheimer disease),^13^ and disruption of functional network connectivity evaluated with magnetic resonance imaging.^14^ However, despite many observations on vascular dysfunction and cognitive decline in patients with cSVD, little attention has been focused on protecting vascular physiology to improve cognitive function.

Nimodipine, a central nervous system-selective dihydropyridine calcium channel blocking agent, has been widely used in the clinic as a vasodilating agent to reduce vasospasm after subarachnoid hemorrhage. Nimodipine is highly lipophilic compared with nifedipine and other dihydropyridine calcium channel–blocking agents,^15^ and its permeability across the blood-brain barrier increases its accessibility to vascular components outside the endothelial tight junctions, including pericytes and vascular smooth muscle cells. Nimodipine is well tolerated, and it has been shown to be beneficial in a range of neurological diseases.^16^ The drug is also of some benefit in vascular and other dementias,^17^ but the mechanisms are uncertain and the clinical benefits are often small.

We have found significant protection by nimodipine in animal models of multiple sclerosis,^18^ and here we investigate whether chronic treatment with nimodipine protects vascular and cognitive function in the spontaneously hypertensive stroke-prone rat (SHRSP), a widely used animal model of cSVD.^19^

## METHODS

**Figure 1.**
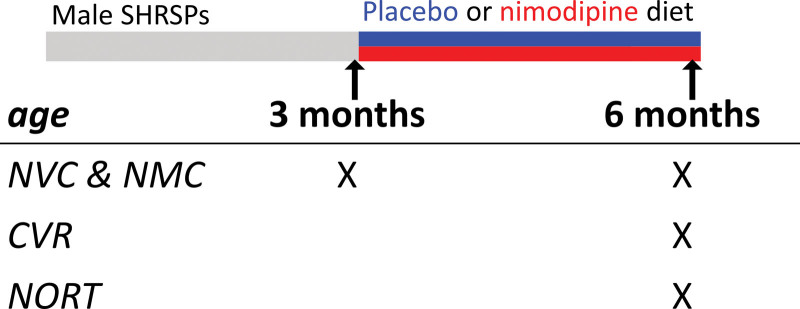
**A brief illustration of the experimental design.** CVR indicates cerebrovascular reactivity; NMC, neurometabolic coupling; NORT, novel object recognition test; NVC, neurovascular coupling; and SHRSP, spontaneously hypertensive stroke-prone rat.

**Figure 2.**
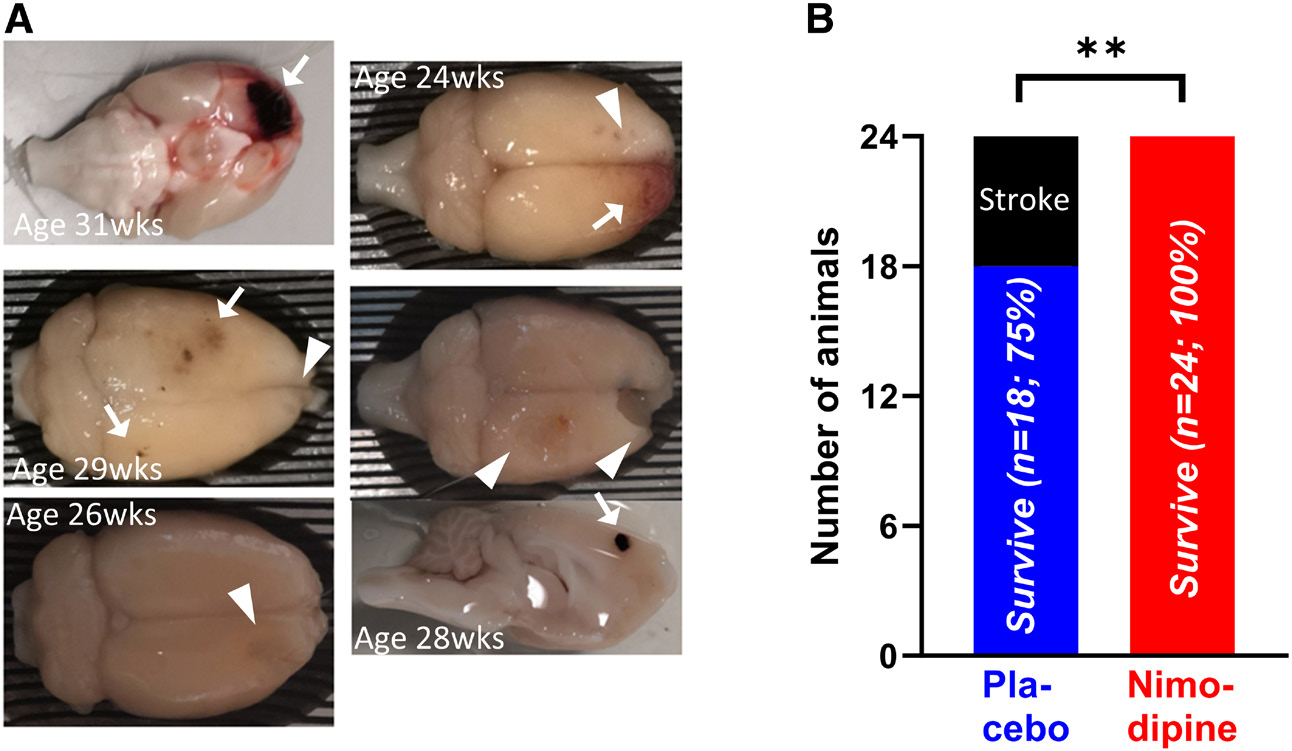
**Some animals in the placebo group experienced a stroke, but no animals in the nimodipine group. A**, Some animals in the placebo group, but not in the nimodipine group, showed severe distress, associated with ischemic (white arrowhead) and hemorrhagic (white arrow) stroke lesions in the brain. **B**, The incidence of adverse events was significantly higher in the placebo group compared with the nimodipine group. Red: nimodipine; n=0/24 adverse outcome. Blue: placebo; n=6/24 adverse outcome. Statistical significance was determined by χ^2^, **P<0.01.

**Figure 3.**
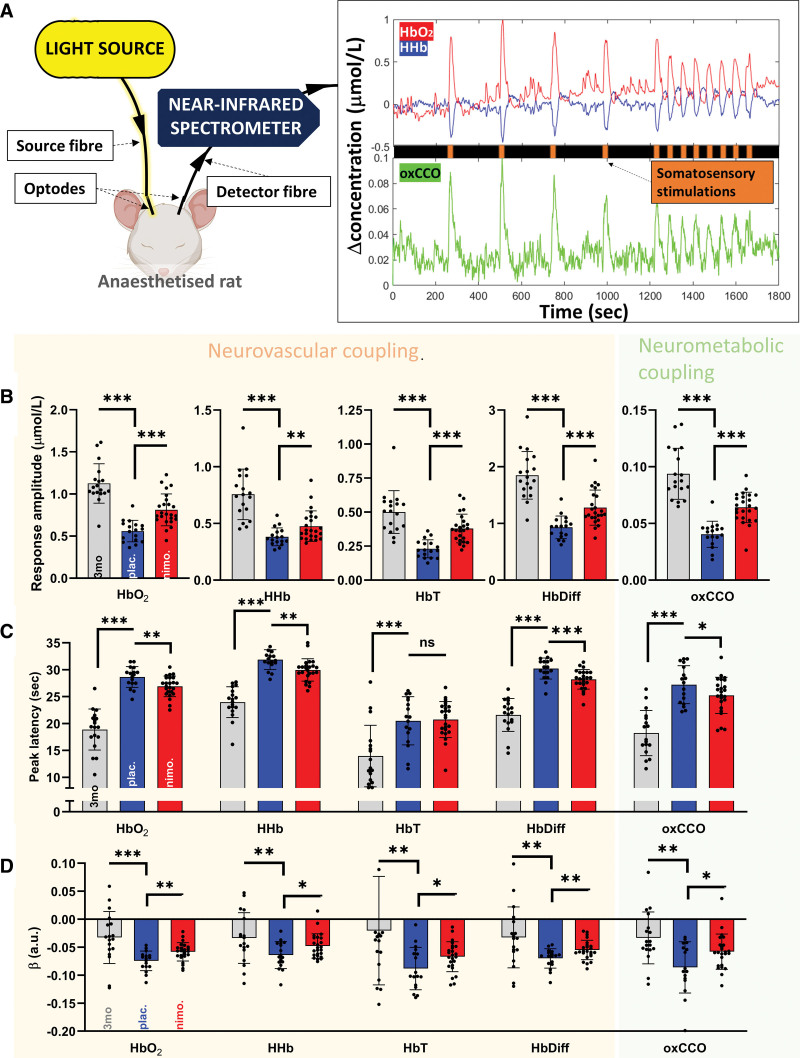
**The data show that, overall, neurovascular coupling (NVC) and neurometabolic coupling (NMC) were impaired as the animals aged but were partially protected by chronic treatment with nimodipine. A**, NVC and NMC were assessed by recordings from the cortex showing changes in oxygenation and metabolism upon somatosensory stimulation, using our customized broadband near-infrared spectroscopy system. In the placebo group (gray bars; **B** through **D**), both NVC (indicated by changes in oxyhemoglobin [Hbo_2_]; deoxyhemoglobin [HHb]; total hemoglobin [HbT]; and difference in hemoglobin [HbDiff]), and NMC (indicated by changes in oxCCO [oxidized cytochrome-c oxidase]) showed high response amplitude (**B**), short peak latency (**C**), and low vulnerability to habituation upon repeated stimulation (**D**) at 3 months of age, but these measures were significantly impaired at 6 months of age (blue and red bars). However, response amplitude (**B**), peak latency (**C**), and vulnerability to habituation (**C**) were all significantly protected in the nimodipine group (red), compared with the placebo group (blue). Gray: animals at 3 months of age; n=18. Red: nimodipine; n=24. Blue: placebo; n=17. Bar plots are indicated as mean±SD. Statistical significance was determined by independent *t* test. ns indicates not significant. *P<0.05, **P<0.01, ***P<0.001.

**Figure 4.**
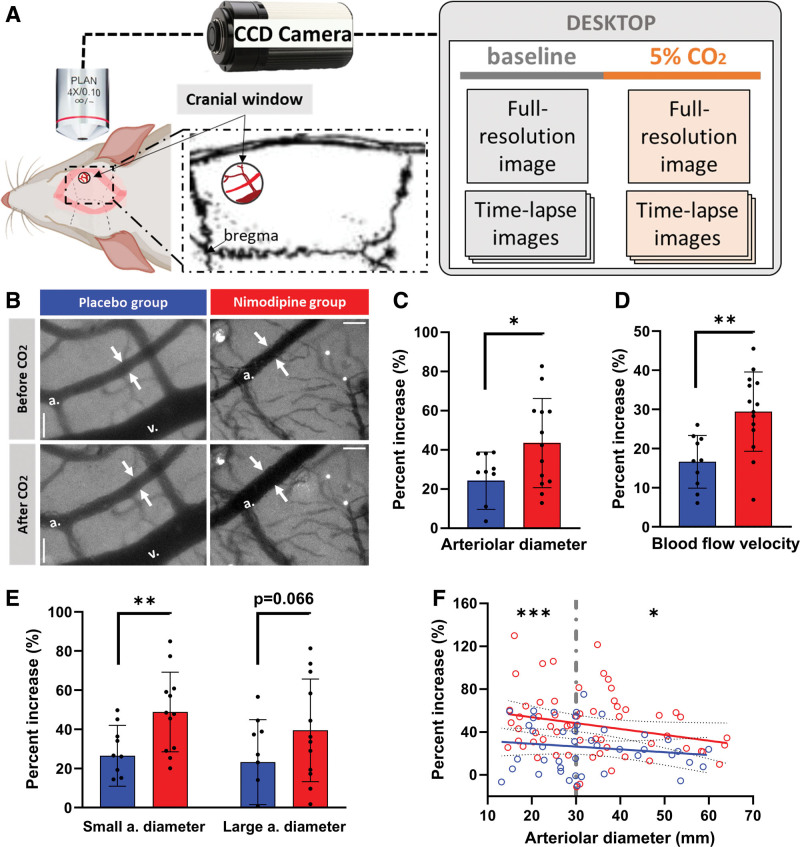
**Animals in the nimodipine group showed better cerebrovascular reactivity (CVR) upon CO_2_ stimulation. A**, CVR was assessed by the dilation of arterioles and increase in venular blood flow velocity upon CO_2_ stimulation, as viewed through a cranial window. **B**, Representative images of cortical vasculature before and after CO_2_ stimulation in both groups. The cortical arteriole (white arrows) of an animal in the nimodipine group showed a larger dilation after CO_2_ compared with that of an animal in the placebo group, while the diameter of venules in both groups remained unchanged. Scale bar=100 µm. **C**, The percentage increase in arteriolar diameter was significantly higher in the nimodipine group, corresponding with (**D**) a significantly higher percentage increase in venular blood flow velocity, indicating a better CVR response. The protective effect of nimodipine treatment on the reactivity of arterioles upon CO_2_ stimulation was more significant in small arterioles with diameter <30 µm, indicated by both (**E**) animal-level and (**F**) vessel-level comparisons. Red: nimodipine; n=14. Blue: placebo; n=10. Bar plots are indicated as mean±SD. Statistical significance was determined by independent *t* test. CCD indicates charge-coupled device; and ns, not significant. *P<0.05, **P<0.01, ***P<0.001.

**Figure 5.**
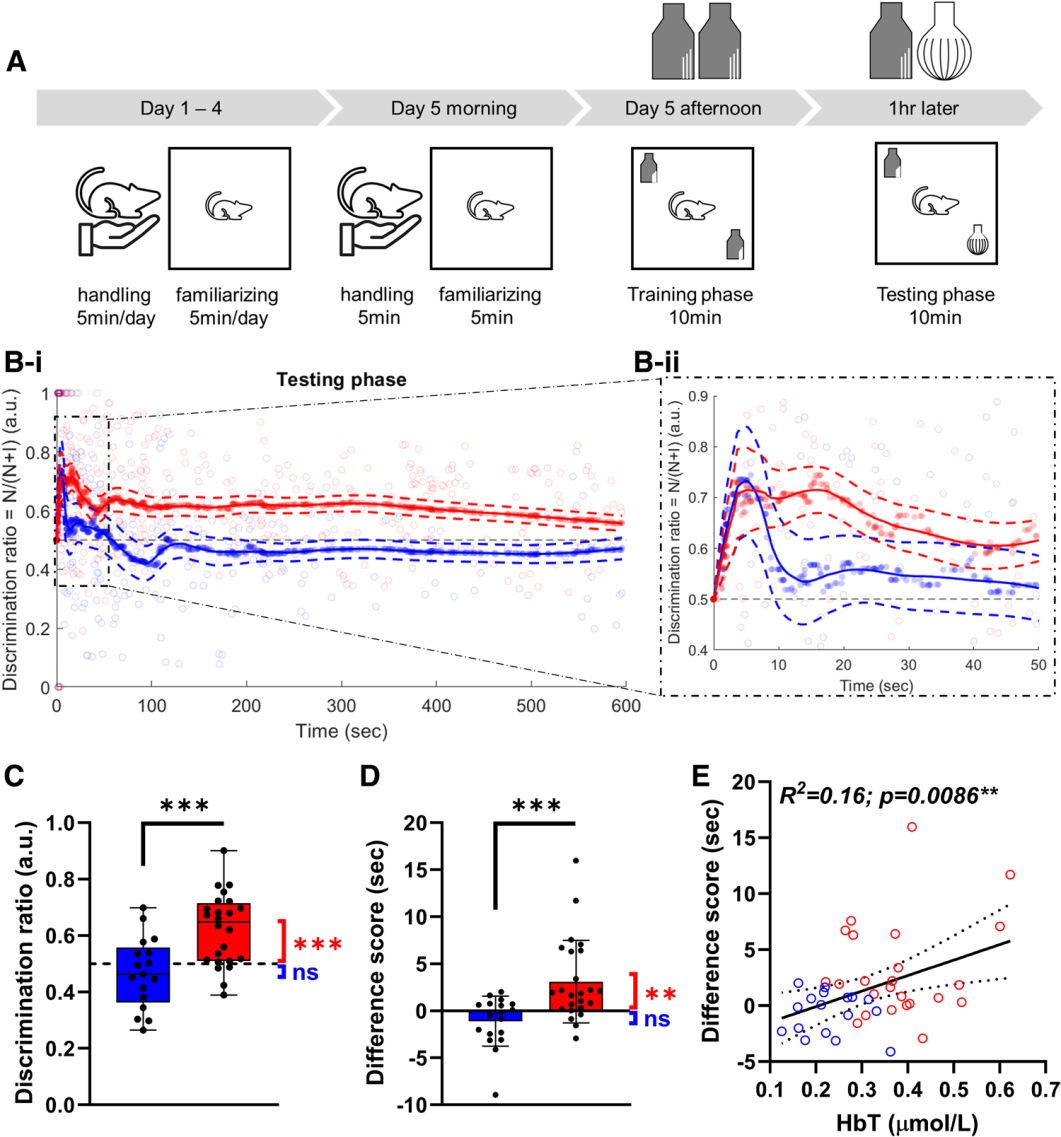
**Animals treated with nimodipine showed better cognitive function, correlating with better vascular function. A**, Cognitive function was evaluated with the novel object recognition test, consisting of 5 consecutive days of handling, a 10-minute training phase and a 10-minute testing phase. **Bi** and **Bii**, The discrimination ratio was plotted for both groups across the 10-minute testing phase, which showed distinct differences between the groups. After 5 minutes of the initiation of the testing phase, the (**C**) discrimination ratio and (**D**) difference score were both significantly higher in the nimodipine group compared with the placebo group. Furthermore, both parameters were significantly higher than reference levels (ie, 0.5 for discrimination ratio and 0 for difference score) in the nimodipine group, but not in the placebo group. **E**, Cognitive function, indicated by the difference score, was significantly correlated with neurovascular coupling, represented by the response amplitude of HbT. Red: nimodipine; n=24. Blue: placebo; n=18. Bar plots are indicated as mean±SD; box plots as minimum to maximum. Statistical significance was determined by independent *t* test and linear regression. ns indicates not significant. *P<0.05, **P<0.01, ***P<0.001.

The authors declare that all supporting data are available within the article and its Supplemental Material. Male SHRSPs were bred in-house and maintained in a 12-hour light/dark cycle, with food and water ad libitum. The animals (see Figure 1) were randomly allocated at 3 months of age to either receive a control diet (placebo group), or a diet containing 200 mg/kg nimodipine (Ssniff Spezialdiäten GmbH, Germany; nimodipine group). Dosing from 3 months was chosen because the pathology of cSVD is usually not fully developed at this time in SHRSPs,^20^ mimicking a clinical scenario where treatment starts when patients show risk factors of cSVD but not yet severe pathology. Both diets continued for 3 months, during which the animals were daily monitored for any abnormal behavior or distress that may indicate a stroke. Affected animals were promptly culled (see Figure 2), and so were excluded from blood pressure measurement, and from study evaluating vascular and cognitive function (next). Observations were made regarding NVC and neurometabolic coupling (NMC) when the animals were 3 months of age, and at 6 months of age, that is, after 3 months on diet, using noninvasive broadband near-infrared spectroscopy system^21^ developed in-house and illustrated in Figure 3A. The peak response amplitude, peak latency, and habituation upon repeated stimulation were quantified with the broadband near-infrared spectroscopy recordings of oxyhemoglobin, deoxyhemoglobin, hemoglobin difference (that is, oxyhemoglobin-deoxyhemoglobin), and hemoglobin total (that is, oxyhemoglobin+deoxyhemoglobin), as well as oxCCO (oxidized cytochrome-c oxidase).^22^ Vascular function was also evaluated by monitoring CVR upon CO_2_ stimulation, in a proportion of animals after 3 months on diet, through an exposed cranial window (Figure 4A). Arteriolar diameter and blood flow velocity were quantified before and after CO_2_ stimulation, and the extent of increase in both parameters was used to indicate CVR. As a measure of cognitive function, the novel object recognition test^23^ was performed after 3 months of diet (Figure 5A). The novel object recognition test was conducted before the aforementioned experiments on vascular function to avoid any effect of anesthesia or surgery on cognitive performance. The time each animal spent interacting with novel and familiar objects was quantified, from which the difference score (t_novel_-t_familiar_) and the discrimination ratio (t_novel_/(t_novel_+t_familar_)) were calculated. All experiments (Figure 1) were performed in accordance with the UK Home Office Animals (Scientific Procedures) Act (1986). The animal study protocol follows the ARRIVE 2 guidelines (Animal Research: Reporting of In Vivo Experiments)^24^ and was approved by the Ethics Committee of University College London and the UK Home Office. Please refer to the Supplemental Material for detailed methods.

## RESULTS

### Chronic Treatment of Nimodipine Reduces Incidence of Stroke in SHRSP

Animals in both placebo and nimodipine groups exhibited elevated systolic blood pressure (placebo: 226.3±12.9 mm Hg; nimodipine: 204.9±11.9 mm Hg), which was greatly above the normal range (around 150 mm Hg; see study by Kaiser et al^19^). Several animals (n=6) in the placebo group showed signs of seizure, or distress, namely hunching, reduced movement, and aggressiveness upon handling, at 17, 24, 26, 28, 29, and 31 weeks of age and were promptly terminated. Gross examination of the brains at postmortem revealed ischemic or hemorrhagic lesions (Figure 2A). No animal in the nimodipine group showed any abnormal behavior when alive, or any visible lesions upon gross examination after termination, resulting in a significantly better outcome for animals in the group treated with nimodipine (Figure 2B; *P*=0.009).

### Nimodipine Preserves Neurovascular and Neurometabolic Coupling

Vascular function was assessed by monitoring the NVC and NMC in both groups of animals at 3 and 6 months of age (Figure 3B through 3D; Table S1). The highest response amplitude (Figure 3B) and the shortest peak latency (Figure 3C) in NVC (oxyhemoglobin, deoxyhemoglobin, hemoglobin difference, and hemoglobin total) and NMC (oxCCO) were observed at 3 months of age. If left untreated, the animals exhibited a progressive decline in NVC and NMC with age, indicated by decreased amplitude and prolonged latency in the 6-month-old placebo group. This decline was significantly less pronounced in the group treated with nimodipine. Upon repeated stimulation with short intervals, the amplitude of the responses typically decreases (as seen in Figure 3A), which we term as habituation. Habituation was least prominent in the 3-month-old group, as indicated by a beta value closest to 0, and it became more prominent with age (Figure 3D). Animals treated with nimodipine were significantly protected from habituation.

### Nimodipine Preserves Cerebrovascular Reactivity

CVR upon CO_2_ inhalation was assessed as a measure of vascular function in a proportion of the animals from both groups (placebo n=10; nimodipine n=14) at the end of the study (Figure 4A). Dilation in cortical arterioles upon CO_2_ stimulation was observed in all animals (Figure 4B), but the percentage increase in arteriolar diameter was significantly larger in the nimodipine group compared with the placebo group (Figure 4C; 24.21±14.63% versus 43.45±22.76%; *P*=0.015). The increase in diameter correlated with a greater increase in blood flow velocity measured in the venules (Figure 4D; 16.63±6.27% versus 29.44±10.14%, *P*=0.001). As indicated by the trend in linear regressions in Figure 4F, the effect of nimodipine in protecting arteriolar reactivity was more prominent in smaller arterioles. When subgrouping arterioles into those with small diameter (<30 µm) versus large diameter (≥30 µm), the protective effect of nimodipine was significant only in the small arterioles (Figure 4E (animal-level comparison; small arterioles: 26.51±15.55% versus 48.87±20.36%; *P*=0.005; large arterioles: 23.25±21.68% versus 39.52±26.20%; *P*=0.066) and Figure 4F (vessel-level comparison; small arterioles: 25.79±22.62% versus 52.46±28.34%; *P*<0.001; large arterioles: 25.60±21.50% versus 42.03±29.69%; *P*=0.017).

### Protection in Vascular Function Is Associated With a Preservation of Cognitive Function

The changes in discrimination ratio of both the placebo and nimodipine groups across 10 minutes of testing phase are shown in Figure 5B through 5I. Animals in both groups showed an increase in the discrimination ratio during the first minute of the testing phase (Figure 5Bii), indicating that the animals were initially attracted to the novel object. However, as the testing phase went on, the discrimination ratio of the placebo group dropped to showing no particular interest, whereas the nimodipine group continued to show a greater interest in the novel object (Figure 5Bi), suggesting that they recognized it as being novel. We chose the first 5 minutes (300 seconds) as a representative time period for quantification of the test phase,^23^ which showed that the nimodipine group had significantly higher discrimination ratio (Figure 5C; 0.46±0.12 versus 0.62±0.12; *P*<0.001) and difference score (Figure 5D; −1.10±2.65 versus 3.09±4.39 seconds; *P*<0.001) compared with the placebo group. Furthermore, the discrimination ratio and difference score were not different from their reference level (0.5 and 0, respectively) in the placebo group (discrimination ratio: *P*=0.96; difference score: *P*=0.08) but were significantly higher than reference levels in the nimodipine group (discrimination ratio: *P*<0.001; difference score: *P*=0.002). Cognitive function, as indicated by the difference score, was significantly correlated with vascular function, as represented by the response amplitude of hemoglobin total (Figure 5E; *P*=0.009).

## DISCUSSION

The study has investigated the therapeutic value of chronic oral administration of nimodipine in SHRSP, an animal model of cSVD. The results show that the treatment provides protection from 2 of the major clinical consequences of cSVD, namely stroke and cognitive deficits.^25^ The protection of cognitive function is associated with the preservation of NVC and CVR, suggesting that dynamic vascular function is a therapeutic target for vascular cognitive impairment and dementia.

Impaired vascular function, represented by decreased NVC and CVR, has been extensively reported in animal models of cSVD^26^ including SHRSP,^27^ and in patients with various forms of cSVD.^28^ Our findings support these observations by revealing that upon somatosensory stimulation, the changes in cerebral blood volume (hemoglobin total) and tissue oxygenation (oxyhemoglobin, deoxyhemoglobin, and hemoglobin difference) are reduced and delayed as animals advance from 3 to 6 months of age. More importantly, using our broadband near-infrared spectroscopy system, we have been able not only to detect the reduction in vascular function but also to show a parallel reduction in mitochondrial function, as revealed by measurement of oxCCO. It is reasonable to predict that the lack of adequate mitochondrial function will result in impaired neuronal function, and it follows that the reduced vascular and mitochondrial function provides a sufficient explanation for the cognitive decline observed in both animal models and patients.

The findings show that NVC and NMC, together with CVR, can be partly protected by chronic treatment with nimodipine. We recognize that anesthesia, including isoflurane anesthesia, can influence the physiological properties of the vasculature, but the same anesthesia was employed for both experimental and control groups, reducing the impact of any such effects. The effects of nimodipine on vascular function have received little attention, but our results are consistent with the available observations showing preservation of NVC by nimodipine in spreading depolarization.^29,30^ In both of these studies, acute administration of nimodipine, either directly into the parenchyma through a cranial window or via intraperitoneal injection, showed a protective effect on the dynamic vascular function. It is encouraging that our findings show the feasibility of achieving chronic protection simply by oral administration of the drug.

The preservation of dynamic vascular function was found to be associated with protection of cognitive function in the same batch of SHRSP. The cognitive protection is in agreement with previous studies showing the beneficial effects of acute or subacute administration of nimodipine in animal models of ischemic stroke, for example,^31^ and by chronic (52 weeks) treatment^32^ of patients with vascular dementia. The current findings suggest that the protection of cognitive function is at least partly dependent on the preservation of dynamic vascular function.

We believe the current study is the first to examine the beneficial effects of chronic nimodipine treatment initiated at an early stage of cSVD, revealing a protection from cognitive decline and a preservation of vascular function. Previous studies have shown that subacute (3 weeks) or chronic (10–20 weeks) treatment with nimodipine starting in older SHRSP (eg, 40 weeks of age) achieves a similar pattern of protection as in our study of younger animals, namely an improvement in survival, and a preservation of cognitive function and microvascular integrity^33–35^ However, the mechanism(s) responsible for the protection remain unclear, but it is notable that nimodipine reduces blood pressure, as in our study, and a number of studies have implicated hypertension as a risk factor for pathologies related to cSVD. Indeed, some antihypertensive treatments have the beneficial effects of reducing the microanatomical changes in spontaneously hypertensive rats,^36^ and also reducing cSVD pathologies.^37^ Antihypertensives can delay cognitive impairment^37^ and reduce the risk of dementia in patients,^38,39^ but the beneficial effects are not ubiquitous. Thus, a clinical trial managing cardiovascular risks for over 6 years resulted in a significantly reduced incidence of hypertension but not of all-cause dementia.^40^ Similarly, lowering blood pressure over 11 years in young patients with recent lacunar stroke also did not protect cognitive function.^41^ Furthermore, some antihypertensive drugs lower the risk of Alzheimer disease and related dementias, but others do not.^42^

Against this background, it is notable that treatment with dihydropyridine calcium channel–blocking agents such as nimodipine is widely beneficial. Nimodipine protects against the consequences of subcortical vascular dementia,^32^ and administration 7 to 14 days after cerebral infarction for 3 months results in memory improvement,^43^ perhaps partly achieved by protection from spreading depolarization and consequent spreading ischemia.^16^ Indeed, use of dihydropyridine calcium channel–blocking agents improves cognitive performance independently of blood pressure level, suggesting a specific neuroprotective effect of this pharmacological class.^44^ It is therefore noteworthy that apart from reducing blood pressure, nimodipine is directly protective to neurons,^16^ and anti-inflammatory effects in reducing astrocyte and microglial activation,^16^ with protective effects on pericytes and oligodendrocytes.^16,45^ Even in vitro, where effects on blood flow are not involved, nimodipine still shows cell type–independent suppression of stress-dependent apoptosis,^46^ and protection of tissue slices^47^ and the neurovascular unit.^48^ In summary, it is likely that the mechanisms underlying the neuroprotective effects of nimodipine are multifactorial.

Interestingly, it appears that nimodipine has particular benefits that are not necessarily shared by other dihydropyridine calcium channel–blocking agents.^47^ Thus, the protective effects of nimodipine are not adequately reproduced by nifedipine in slice cultures,^49^ or by nicardipine in a systematic review.^50^ Indeed, although nimodipine is used to improve outcome in subarachnoid hemorrhage on the basis that it avoids delayed vasoconstriction, the actual mechanism of action is now being re-evaluated since other interventions that successfully target angiographic vasospasm have not improved outcomes.^51^ In fact, we anticipate that the observed protection of vascular and cognitive function by nimodipine is at least partly through a preservation of cellular components of the neurovascular unit, even behind the blood-brain barrier due to the fact that nimodipine is highly lipophilic. Nimodipine improves endothelial function (blood brain barrier integrity) after anoxia-reoxygenation in an in vitro study where effects on blood flow are not involved.^48^ Indeed, drugs protecting endothelial cells preserve CVR^52^ and cognitive function^53^ in patients with lacunar stroke, without affecting blood pressure. While the cellular mechanism of the therapeutic effect of nimodipine in the current study still needs investigating, the findings suggest a pathway where vascular dysfunction contributes to cognitive decline, and nimodipine protects vascular function.

## CONCLUSIONS

The results show that chronic treatment with nimodipine protects from strokes and the decline in both cognition and vascular function that normally occur during prolonged disease in cSVD and its SHRSP model. Further, the findings indicate that the decline in cognitive function is partly due to a decline in dynamic vascular functions and that nimodipine protects dynamic vascular function. Our findings suggest a rationale for further testing of nimodipine in randomized trials in patients with cSVD and for further validation of dynamic vascular function as an early biomarker not only for vascular cognitive impairment and dementia but also potentially for other types of dementia and neurodegenerative diseases.

## ARTICLE INFORMATION

### Acknowledgments

The authors gratefully acknowledge the expert advice of Dr Liam Browne regarding the behavioral assessment (novel object recognition test) of cognitive function.

### Sources of Funding

The research was supported by funding to Dr Smith from the Multiple Sclerosis Society (United Kingdom), the International Progressive Multiple Sclerosis Alliance, and the Fondation Leducq. Dr Werring has received grant funding from the Stroke Association and British Heart Foundation.

### Disclosures

Dr Werring has received speaking honoraria from Bayer; speaking and chairing honoraria from Alexion Pharmaceuticals Inc and Novo Nordisk; and consultancy fees from Alnylam Pharmaceuticals, Bayer, and Novo Nordisk. Dr Tachtsidis is the CEO and founder of Metabolight Ltd.

### Supplemental Material

ARRIVE guideline checklist

Supplemental Materials and Methods

Table S1

## Supplementary Material
