# Supplementary material for: Nimodipine Protects Vascular and Cognitive Function in an Animal Model of Cerebral Small Vessel Disease
Source: Stroke. 2024 Jun 11;55(7):1914–22. doi: 10.1161/STROKEAHA.124.047154 (PMC11251505; doi:10.1161/STROKEAHA.124.047154)
Supplement: Supplementary file 2 [file str-55-1914-s002.pdf]

SUPPLEMENTAL MATERIAL

Nimodipine protects vascular and cognitive function in an animal model of cerebral small vessel disease

Zhiyuan Yang,[a*] Frédéric Lange,[b] Yiqing Xia,[a] Casey Chertavian,[a] Katerina Cabolis,[a] Marija Sajic,[a] David J. Werring,[c] Ilias Tachtsidis,[b] and Kenneth J. Smith[a*]

[a] Department of Neuroinflammation, UCL Queen Square Institute of Neurology, University College London, London, UK; [b] Department of Medical Physics and Biomedical Engineering, University College London, London, UK; [c] Stroke Research Centre, Department of Brain Repair and Rehabilitation, UCL Queen Square Institute of Neurology, University College London, London, UK.

*Co-corresponding authors

Animals and study design

Male SHRSPs (commonly used as a model of cSVD) were bred in house and housed with a 12 hour light/dark cycle, and food and water *ad libitum*. The animals were randomly allocated at three months of age to either receive a control diet (placebo group), or a diet containing 200mg/kg nimodipine (Ssniff Spezialdiäten GmbH, Germany) (nimodipine group). The consumption of nimodipine diet by each animal provides a dose of ~10mg per kg body weight, per day. Dosing from three months was chosen because the pathology of cSVD is usually not fully developed at this time in SHRSPs, mimicking a clinical scenario where treatment starts when patients show risk factors of cSVD, but not yet severe pathology. Both diets continued for three months, during which the animals were daily monitored for any abnormal behaviour or distress that may indicate a stroke. Affected animals were promptly culled (see **Figure 2**), and so were excluded from blood pressure measurements and from further study evaluating vascular and cognitive function (below). Blood pressure was measured in the 6-month-old animals using an indirect tail-cuff method (CODA High Throughput System; Kent Scientific, UK), and systolic pressure was determined as an average of 10 cuff inflation measurements. All experiments (**Figure 1**) followed the ARRIVE 2 guidelines, and were performed in accordance with the UK Home Office Animals (Scientific Procedures) Act (1986). The animal study protocol was approved by the Ethics Committee of University College London and the UK Home Office.

Neurovascular and neurometabolic coupling

Animal preparation and stimulation protocol

Observations were made regarding neurovascular coupling (NVC) and neurometabolic coupling (NMC) when the animals were 3 months of age, and at 6 months of age, i.e. after 3 months on diet, using non-invasive broadband near infrared spectroscopy (bNIRS) system developed in-house, as illustrated in **Figure 3 a**. Animals were anaesthetized with 2% isoflurane in room air, with body temperature maintained at 37°C using a homeothermic heating pad (Harvard Apparatus, Cambridge, UK), and vital signs monitored with a pulse oximeter (MouseOx; STARR Life Sciences, Oakmont, USA) positioned on the foot. The head was stabilized using a customized stereotaxic frame, and somatosensory cortical regions were marked 4mm lateral and 0.2mm rostral to bregma, on both sides.

After shaving the head, the source and detector fibres of the bNIRS system were placed in close contact with the skin over the right and left somatosensory cortical regions of the fore paws, respectively. The source fibre was connected to a halogen light source (HL-2000-HP), and detector fibre to a customized miniature spectrometer (Ventana VIS-NIR). Particular wavelengths of light passing through the brain from the source to the detector fibre are absorbed by tissues and blood with distinct extinction coefficients. The absorption alters the emitted spectrum detected by the spectrometer. Based on this alteration, our algorithm is able to

calculate changes in the concentration of not only oxyhaemoglobin (HbO_2) and deoxyhaemoglobin (HHb), but also oxidized cytochrome-c oxidase (oxCCO; i.e., complex IV) in the mitochondria, thus providing evaluation on both cerebral haemodynamics and metabolism.

To perform somatosensory stimulation, a set of custom-made needle electrodes were inserted percutaneously between 1st and 2nd digit and between 3rd and 4th digit of both fore paws. The somatosensory cortexes were activated by customized trains of electrical stimulation at an intensity of 4mA lasting 500μs, in a train of 15sec at 3Hz using an isolated current stimulator (DS3, Digitimer, USA). Two types of stimulation were performed with different intra-stimulation intervals. The first type of stimulation consisted of four trains of 15sec stimulations with 225sec of intervals between two trains. The second type of stimulation was performed 225sec after the fourth stimulation, which consisted of eight trains of 15sec stimulations with 45sec of intervals. The first type of stimulation, i.e., stimulations with long interval, was intended to evaluate the overall NVC and NMC. And the second type of stimulation, i.e., stimulations with short interval, was to assess the "habituation", namely the decline in the NVC and NMC upon repeated stimulations.

Data quantification

Five parameters were taken into account, namely HbO_2, HHb and oxCCO directly measured from the system, as well as HbDiff (i.e. the mismatch between oxy- and deoxy-haemoglobin; HbO_2-HHb) and HbT (i.e. the total arterial+venous mixed blood volume; HbO_2+HHb) calculated to indicate overall haemodynamic response. For the long-interval stimulations, four responses were averaged to locate the peak of response, from which the peak response amplitude and the leak latency were identified for each parameter. For the short-interval stimulations, peak response amplitudes were generated for each of the eight responses. Then a linear regression was performed between the sequence of stimulation (i.e., 1 to 8) and the peak amplitudes normalized to that of the first stimulation. The beta value was retrieved from the linear regression to evaluate the extent of decrease in peak amplitude upon repeated stimulation.

Cerebrovascular reactivity

Surgical preparation and microscopy imaging

Vascular function was also evaluated with cerebrovascular reactivity (CVR) upon CO_2 stimulation, in a proportion of animals after 3 months on diet (**Figure 4 a)**. Animals were anaesthetized with a ketamine (Ketamidor; Chanelle Pharma, Ireland) and xylazine (Rompum; Bayer, Germany) mixture (1ml/kg BW) injected intra-peritoneally (i.p.). Stabilization of head, maintenance of body temperature and monitoring of vital signs were conducted as described previously. A craniotomy was performed at the somatosensory cortical region on the right hemisphere to create a 3mm diameter window for imaging. The animals were then transferred under a fluorescent microscope (Zeiss, Germany) mounted with a CCD camera (QImaging, USA) and illuminated by an X-Cite lamp (Excelitas, Canada). First, a full-resolution image of the cortical vasculature at baseline condition was taken with 200ms exposure time under a 4x objective (Olympus, Japan) through a FITC filter cube (Filter Set 10; Zeiss, Germany), using Micro-Manager software (μManger, USA). Then, the animals were injected with 100ul of diluted fluorescent microspheres (1:1000 in saline; F8827; Invitrogen) through a pre-inserted i.v. catheter in the femoral vein, and a time-lapse of 2000 images was taken with 10ms exposure time. After injected into the blood stream, the fluorescent microspheres would flow with the blood, thus the speed of the microspheres could represent blood flow velocity in the same vessel, if averaged from a sufficient number of measurements. After images were taken at baseline condition, the animals were administered with 5% CO_2 in room air for 3min, and another full-resolution image, as well as 2000 time-lapse images were taken at the same location to reveal vasculature and blood flow upon CO_2 stimulation.

Data quantification

Arterioles and venules were identified by the vessel diameter and the direction of the blood flow within the vessel. Arteriolar diameter was measured with ImageJ (NIH, USA) in the full-resolution images at three to

six different branches at the same place before and after CO_2 stimulation to represent the extent of dilation. Each diameter reading was recorded as an average of five measurements. The percent increase of the arteriolar diameter was calculated as the absolute increase in diameter normalized to the diameter at baseline condition.

Blood flow velocity was measured in cortical venules, due to that arteriolar blood flow velocity is too fast to be accurately measured with our experimental setting. The velocity of fluorescent microspheres was quantified with a kymograph method using the Multi-Kymograph plugin in ImageJ which tracks the fluorescent microspheres as they flow along a vessel. Over 40 microspheres were tracked for each set of time-lapse images, and the velocities of the microspheres were averaged to represent blood flow velocity. For each pair of time-lapse images taken at baseline condition and after CO_2 stimulation, the blood flow velocities of a same venule before and after CO_2 stimulation were acquired. The percent increase in the venular blood flow velocity was calculated as the absolute increase in the blood flow velocity normalized to the blood flow velocity at baseline condition.

Novel object recognition test

Novel object recognition test (NORT) is commonly used to evaluate learning and memory ability of rodents, based on their natural behaviour of exploring novel things. The test (**Figure 5 a**) was performed when the animals were 6 months of age (i.e., after 3 months on diet), and it was conducted before the aforementioned experiments on vascular function to avoid any effect of anaesthesia or surgery on cognitive performance. The NORT was conducted in an arena made from a black acrylic box (50x50x50cm) located in a quiet room in the animal facility. The animals were handled for 5 minutes and were put into the box to acclimatise for another 5 minutes, for five consecutive days before testing. On the day of testing, a 10-minute training phase was first performed, when each animal was allowed to explore two identical objects placed diagonally in the arena. An hour later, one of the identical objects was randomly chosen to be removed and replaced with a novel object, and a 10-minute testing phase was then performed. Both phases were video-recorded, and interaction time of each animal spent with each object was manually recorded by a researcher blinded to group allocation. Valid interaction was identified as nose touching and sniffing the object, with whiskers moving. To evaluate the extent of interaction, the difference score (t_{novel}-$t_{familiar}$) and the discrimination ratio ($t_{novel}/(t_{novel}+t_{familar})$) were calculated and compared between the placebo and nimodipine groups.

Statistics

Data quantification and analysis were performed blinded to group allocation. Differences between groups were compared using Chi-square, independent t-test and linear regression with Graphpad Prism and Matlab. Results are presented as mean ± standard deviation (SD) in the text and graphs, unless otherwise indicated.

Supplemental table

Table S1. Summary of NVC and NMC in animals at 3 months of age, and in animals at 6 months of age in the placebo and nimodipine group.

	HbO_2	HHb	HbT	HbDiff	oxCCO
**Response amplitude (μmol/L)**					
3-month-old	1.12±0.22	0.75±0.21	0.50±0.15	1.84±0.40	0.093±0.021
Placebo @6m	0.55±0.12	0.38±0.076	0.23±0.064	0.93±0.19	0.040±0.011
Nimodipine @6m	0.81±0.18	0.47±0.13	0.37±0.10	1.27±0.30	0.064±0.013
p values					
3mo vs. placebo@6mo	*<0.001*	*<0.001*	*<0.001*	*<0.001*	*<0.001*
Placebo@6mo vs. nimodipine@6mo	*<0.001*	*0.0068*	*<0.001*	*<0.001*	*<0.001*
**Peak latency (sec)**					
3-month-old	18.87±3.71	23.96±2.79	13.95±5.54	21.58±2.96	18.22±4.07
Placebo @ 6m	28.65±1.86	31.87±1.79	20.50±4.34	30.21±1.89	27.21±3.42
Nimodipine @ 6m	26.90±1.87	29.95±2.03	20.73±3.27	28.20±1.76	25.22±3.28
p values					
3mo vs. placebo@6mo	*<0.001*	*<0.001*	*<0.001*	*<0.001*	*<0.001*
Placebo@6mo vs. nimodipine@6mo	*0.0032*	*0.0021*	*0.42*	*<0.001*	*0.037*
**β (a.u.)**					
3-month-old	-0.032±0.045	-0.033±0.043	-0.020±0.094	-0.032±0.052	-0.033±0.045
Placebo @ 6m	-0.074±0.016	-0.064±0.023	-0.088±0.036	-0.069±0.016	-0.085±0.044
Nimodipine @ 6m	-0.058±0.016	-0.047±0.021	-0.067±0.026	-0.054±0.016	-0.058±0.030
p values					
3mo vs. placebo@6mo	*<0.001*	*0.0094*	*0.0055*	*0.0052*	*0.0011*
Placebo@6mo vs. nimodipine@6mo	*0.0021*	*0.014*	*0.021*	*0.0048*	*0.013*

3mo: 3-month-old animals; HbDiff: difference in haemoglobin; HbO_2: oxyhaemoglobin; HbT: total haemoglobin; HHb: deoxyhaemoglobin; oxCCO: oxidized cytochrome-c oxidase. Results presented as mean±SD.

SUPPLEMENTAL MATERIAL
